# Repair of Unstable Posterior Sternoclavicular Dislocation Using Nonabsorbable Tape Suture and Tension Band Technique: A Case Report with Good Results

**DOI:** 10.1155/2015/750898

**Published:** 2015-11-03

**Authors:** Ekrem Aydın, Turan Cihan Dülgeroğlu, Ali Ateş, Hasan Metineren

**Affiliations:** ^1^Department of Orthopaedics and Traumatology, Dumlupinar University School of Medicine, 43270 Kutahya, Turkey; ^2^Department of Orthopaedics and Traumatology, Karaman State Hospital, 70200 Karaman, Turkey

## Abstract

Posterior sternoclavicular joint dislocation (PSCJD) is quite a rare condition. Nearly half of the closed reduction attempts fail due to various reasons. In this paper, we present a 25-year-old male patient who was admitted to the emergency department in our hospital after having a motor-vehicle accident. It was decided to do PSCJD after physical and imaging studies. Following necessary preparations, closed reduction was attempted with abduction-traction maneuver under general anesthesia; however, adequate stabilization could not be achieved and redislocation was detected during control. Therefore, joint was stabilized with tension band technique using 6 mm polyamide nonabsorbable type suture during open reduction. Painless and complete range of motion in shoulder was achieved at the postoperative 10th week.

## 1. Introduction

Sternoclavicular joint dislocation (SCJD) is quite rare, constituting less than 1% of all dislocations in the body. Posterior sternoclavicular joint dislocation (PSCJD) is even rarer [1]. Although it is mainly observed after high energy trauma, it can also occur during sport injuries or simple falls in few cases [2, 3]. PSCJD can result in many complications, even death [2]. Some of these complications are vascular injury, pressure on trachea, vocal cord paralysis, and brachial plexus injury [3–6]. In children, PSCJD also generally occurs after high energy trauma and is generally together with epiphyseal fracture.

## 2. The Case and the Procedure

A 25-year-old male patient applied to our emergency department after having a motor-vehicle accident. He had intense pain on sternum with movements of the arm and shoulder. He had pain during inspirium and expirium. There were no additional significant injuries.

**Figure 1:**
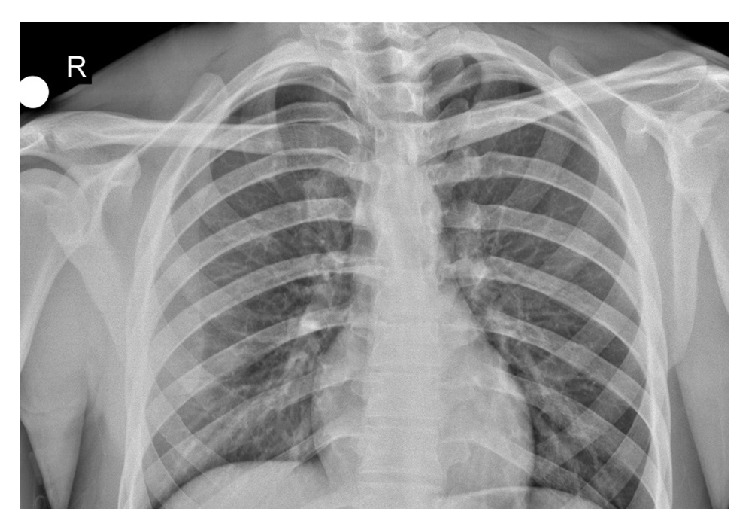
Preop radiographs.

**Figure 2:**
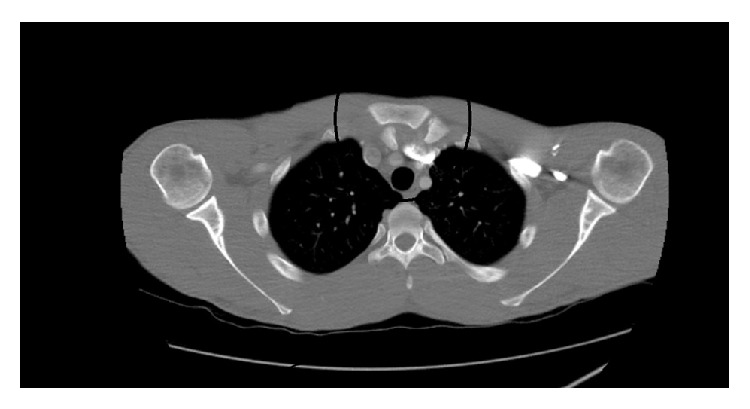
Preop CT.

In his radiographs, clavicle appeared slightly displaced distally (Figure 1). CT revealed that clavicle was apparently displaced posteroinferiorly towards SCJ (Figure 2). After necessary emergent preparations, the patient was admitted to the operation room. Under general anesthesia, the patient was admitted to the operation table in supine position and placing a support between the two scapulas, abduction-traction maneuver was performed. Reduction was confirmed with the help of fluoroscopy. However, it was noticed during shoulder movements for control of stability that the reduction was not stable. Afterwards, it was decided that open reduction and ligament repair should be done. The joint was exposed by making an incision beginning at a point 2 cm away from SCJ towards clavicle side and extending it 4 cm distally along the edge of manubrium sterni. Intra-articular hyaline cartilage appeared dislocated, joint capsule was ruptured from both anterior and posterior sides, clavicular component of the sternocleidomastoid muscle tendon was partially torn.

**Figure 3:**
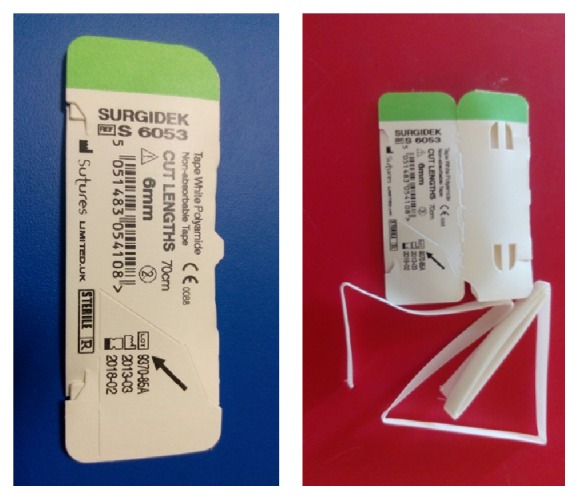
Polyamide nonabsorbable type.

**Figure 4:**
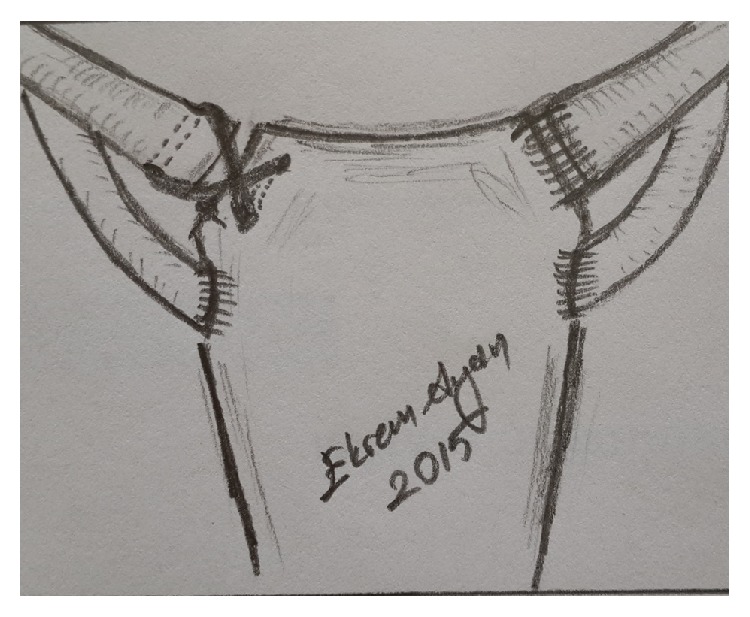
Schematic.

**Figure 5:**
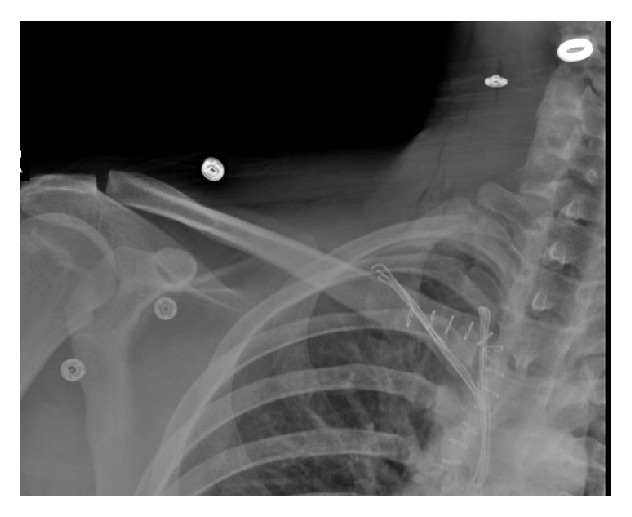
Postop radiographs.

**Figure 6:**
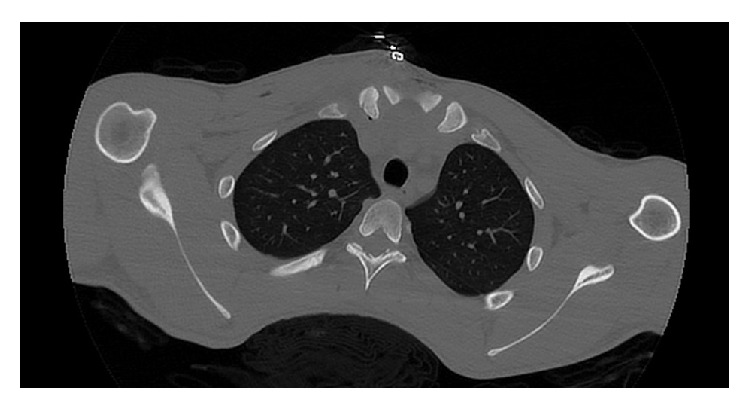
Postop CT.

Two holes were made using 3.2 mm drill at the sternum 1 cm away from the joint surface. And again, using 3.2 cm drill, holes were made at vertical plane 1 cm away from the medial end of clavicle, as to cross both cortices. Six mm polyamide nonabsorbable type sutures passed through these holes and fixated using tension band technique (Figures 3 and 4). Cartilage and joint capsule were repaired as far as possible using 2/0 nonabsorbable suture, and then the torn part of the sternocleidomastoid muscle tendon was sutured onto the capsule. Sternoclavicular joint reduction were evaluated by X-ray and CT images after surgery (Figures 5 and 6). The patient was immobilised by postoperative figure eight bandage and shoulder-arm brace; they were kept for 4 weeks. During control at the postoperative 6th week, the range of motion at the shoulder was complete, but the patient had mild pain at the level of joint. He did not have any pain at the postoperative 10th week. Assessment with ASES shoulder score showed that there was no difference between right and left shoulder movements.

## 3. Discussion

PSCJD may sometimes be overlooked in conventional radiograms; therefore, clinical findings are important for diagnosis. Computed tomography (CT) is gold standard for diagnosis [7, 8]. Clinically, it may be manifested with intense pain, difficulty with deep breathing, and dysphagia. Distal end of the clavicle cannot be palpated, and corner of manubrium sterni can be felt [3]. Considering its proximity to important structures in this area, reduction should be performed as soon as possible. Successful results can be achieved with closed reduction in nearly half of the cases [9]. The most preferred reduction maneuver is to apply abduction-traction maneuver technique to the shoulder while the patient is lying in supine position and the distance between two scapulas is supported with a towel or pillow [10].

Although previously some authors recommended percutaneous Kirschner wire fixation after closed reduction, today it is certainly not recommended because of the possibility of wire migration and penetration to major vessels [8]. Approximately half of the attempts for closed reduction fail due to various reasons. Moreover, serious complications have been reported after closed reductions, which include injuries to trachea and pulmonary artery [4]. For this reason, many authors recommend early open reduction in PSCJD.

Since PSCJ is not a ball-socket type, joint stabilization is provided by intra-articular disk and capsular ligaments. Following dislocation, these ligaments are generally seriously injured, which may lead to insufficiencies after closed reduction as in our case [9, 10]. In their meta-analysis, Tepolt et al. stated that chances of successful closed reduction are low if it is not performed within the first 48 hours [9]. Ngom et al. stated that reduction could be facilitated during closed reduction by pulling clavicle anteriorly with the help of a hooked clamp [2]. After closed reduction, figure eight bandage is applied generally for 6–8 weeks.

Surgery is indicated when closed reduction is unsuccessful and when reduction is lost at the early period [2]. Until today, several open reduction and repair methods have been described with successful outcomes. The classical method is stabilization with local tissue transfer, which was defined by Burrows in 1951 [11]. Since then, several stabilization techniques have been described, including cannulated screw fixation, bridge plate, wire fixation, and repair with allograft or autograft tendon [12]. In our case, tension band technique was used with 6 mm polyamide* nonabsorbable* sutures. Shuler and Pappas had good clinical outcomes after bridge plate application; however, they reported SCJ failure and early joint arthrosis after plate removal due to insufficient tissue healing. The plate should not be removed for at least 3 months [13]. We had favorable results in our case with open reduction in the early period and tension band fixation using 6 mm polyamide nonabsorbable type sutures.

## 4. Conclusion

Reconstruction of posterior sternoclavicular joint dislocation (PSCJD) with 6 mm polyamide nonabsorbable type sutures in tension band pattern through drill holes in the manubrium and clavicle has good results.
